# Adenovirus type 7d outbreak associated with severe clinical presentation, Finland, February to June 2024

**DOI:** 10.2807/1560-7917.ES.2025.30.7.2500061

**Published:** 2025-02-20

**Authors:** Santtu Heinonen, Elina Erra, Richard Lundell, Aino Nyqvist, Pilvi Hepo-oja, Laura Mannonen, Hanna Jarva, Raisa Loginov, Erika Lindh, Leif Lakoma, Petri Kangaspunta, Ilkka Laaksi, Marjaana Pitkäpaasi, Carita Savolainen-Kopra, Simo Nikkari, Hanna Nohynek, Otto Helve, Eeva Ruotsalainen, Niina Ikonen

**Affiliations:** 1 Department of Public Health, Finnish Institute for Health and Welfare (THL), Helsinki, Finland; 2 Department of Pediatrics, University of Helsinki and Helsinki University Hospital, Helsinki, Finland; 3 FVR – Finnish Vaccine Research, Tampere, Finland (current affiliation); 4 Division of Infectious Diseases, Inflammation Center, Helsinki University Hospital, Helsinki, Finland; 5 Epidemiological Operations Unit, City of Helsinki, Helsinki, Finland; 6 Centre for Military Medicine, Finnish Defence Forces, Helsinki, Finland; 7 HUS Diagnostic Center, Department of Clinical Microbiology, Helsinki University Hospital and University of Helsinki, Helsinki, Finland

**Keywords:** adenovirus, type 7d, garrison, military, outbreak

## Abstract

We report an adenovirus outbreak with unusually severe clinical presentation, particularly in military conscripts and their close contacts. During 1 February–30 June 2024, 129 patients with adenovirus infection were hospitalised, 30 were admitted to ICU, 10 required ECMO treatment and six died. Cases consisted of 75 conscripts (58.1%) and 54 civilians (41.9%). Most samples were type 7 (97/108; 89.8%); all 24 sequenced samples were subtype 7d. During 1 August–30 November 2024, 274 additional hospitalised cases were identified from registries.

In late February 2024, the Finnish Institute for Health and Welfare (THL) was notified of two military conscripts hospitalised at the Helsinki University Hospital (HUS) with unusually severe adenovirus infection. Retrospectively, two additional conscripts hospitalised with adenovirus infection in February were identified. Based on this information, an outbreak investigation was initiated. Here, we report the characteristics and outcomes of 129 cases hospitalised with adenovirus infection in Finland between February 1 and 30 June along with the molecular characterisation of the detected adenovirus strains.

## Outbreak investigation

In Finland, clinical microbiological laboratories report positive adenovirus test results to the national infectious diseases register (NIDR) [1]. NIDR does not capture any clinical parameters, outcomes or adenovirus type, and there is no active surveillance system for severe acute respiratory tract infections (SARI) in Finland. 

For this outbreak investigation, we defined a case as ‘a patient hospitalised with laboratory confirmed adenovirus infection irrespective of clinical presentation in Finland after 1 February 2024.’ In order to monitor severe adenovirus infections requiring hospitalisation, we initiated active case detection on 26 February 2024 in all five university hospital regions. We requested the regions to report all hospitalised adenovirus-positive patients to THL and to interview hospitalised civilians about any contacts with conscripts. Interviews were conducted in non-structured manner. To facilitate early detection of a potential increase of severe cases in the general population, we considered any potential contacts with conscripts. Adenovirus testing was based on local guidelines and clinician's discretion. Testing of cases’ contacts for contact tracing purposes was not recommended. 

A task force with members from THL, HUS, the Finnish Defence Forces and specific wellbeing services counties was formed. Other European Union/European Economic Area (EU/EEA) countries were informed about the outbreak through the EpiPulse portal.

## Hospitalised cases

**Figure 1:**
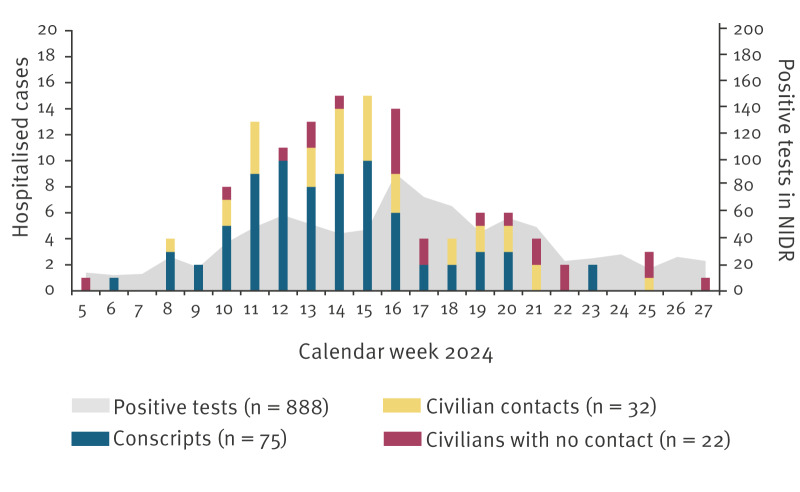
Epidemiological curve of cases hospitalised with adenovirus infection (n = 129) and positive adenovirus tests reported to NIDR (n = 888), Finland, 1 February–30 June 2024

Between 1 February and 30 June 2024 (weeks 5–27), a total of 129 hospitalised cases were reported, including 75 (58.1%) conscripts and 54 (41.9%) civilians. Of the civilians, 32 of 54 had close or possible contact with conscripts: 19 of 32 were close contacts (mostly family members) of conscripts, nine had a possible epidemiological link to conscripts and four were healthcare-associated infections (HAI). (Table 1, Figure 1).

**Table 1:** Characteristics and outcomes of cases hospitalised with adenovirus infection, Finland, 1 February–30 June 2024 (n = 129)

Characteristics	Conscripts n = 75		Civilian contacts^a^ n = 32		Civilians with no contact n = 22		Total n = 129		p^b^
	n	%	n	%	n	%	n	%	
Age in years, median (IQR)	19 (19–20)		48 (23–52)		37 (4–53)		20 (19–33)		< 0.0001
Sex									
Male	72	96.0	20	62.5	13	59.1	105	81.4	< 0.0001
Female	3	4	12	37.5	9	40.9	24	18.6	
**Underlying medical conditions**									
No underlying medical condition	50	86.2	10	38.5	6	40.0	66	66.6	< 0.0001^c^
Non-severe chronic medical condition	8	13.8	6	23.1	1	6.7	15	15.2	
Complex or ≥ 2 chronic medical conditions	0	0	7	26.9	6	40.0	13	13.1	
Immunocompromised	0	0	3	11.5	2	13.3	5	5.1	
Data not available	17		6		7		30		
**Co-detection of other viruses or bacteria**									
No other pathogens	46	64.8	24	82.8	14	70.0	84	70.0	0.21^d^
One additional pathogen	23	32.4	2	6.9	5	25.0	30	25.0	
Multiple additional pathogens	2	2.8	3	10.3	1	5.0	6	5.0	
*Bordetella pertussis*	3	4.2	0	0	0	0	3	2.5	
*Mycoplasma pneumoniae*	5	7.0	2	6.9	0	0	7	5.8	
*Streptococcus pyogenes*	0	0	0	0	1	5.0	1	0.8	
Influenza A	0	0	0	0	0	0	0	0	
Influenza B	1	1.4	0	0	0	0	1	0.8	
Metapneumovirus	3	4.2	0	0	0	0	3	2.5	
Parainfluenza 3 virus	6	8.5	1	3.4	0	0	7	5.8	
Parainfluenza virus (type unknown)	0	0	0	0	2	10.0	2	1.6	
Rhino/enterovirus	3	4.2	3	10.3	3	15.0	9	7.5	
RSV	0	0	0	0	1	5.0	1	0.8	
SARS-CoV-2	1	1.4	1	3.4	0	0	2	1.6	
Seasonal coronavirus	5	7.0	2	6.9	0	0	7	5.8	
Data not available	4		3		2		9		
**Adenovirus type**									
Type 7	60	96.8	28	96.6	9	52.9	97	89.8	< 0.0001^f^
Type 14	0	0	1	3.4	0	0	1	0.9	
Negative^e^	2	3.2	0	0	8	47.1	10	9.3	
Data not available	13		3		5		21		
**Outcome** ^g^									
Normal ward	61	81.3	18	56.3	20	90.9	99	76.7	0.0043^i^
Intensive care unit^h^	14	18.7	14	43.8	2	9.1	30	23.2	
ECMO^h^	3	4.0	7	21.9	0	0	10	7.8	
Death^h^	0	0	5	15.6	1	4.5	6	4.7	

ECMO: extracorporeal membrane oxygenation; IQR: interquartile range; RSV: respiratory syncytial virus; SARS-CoV-2: severe acute respiratory syndrome coronavirus 2.

^a^ Civilian contacts include close contacts of conscripts (n = 19), cases with possible epidemiological link to conscripts (n = 9) and those with healthcare-associated infections (n = 4).

^b^ Kruskal–Wallis test used to compare continuous variables and Chi-square or Fisher’s exact test to compare proportions.

^c^ Comparison between no underlying medical condition vs any underlying medical condition or immunosuppression.

^d^ Comparison between no other pathogens vs one or more additional pathogens.

^e^ PCR typing for adenovirus types 3, 4, 7, 11, 14, 16 and 21 was negative.

^f^ Comparison between adenovirus type 7 vs type 14 or a negative typing result.

^g^ Percentages calculated of all hospitalised cases.

^h^ All ECMO-treated and deceased cases were admitted to the ICU and are also included in the intensive care unit outcome.

^i^ Comparison between normal ward vs intensive care unit.

NIDR: national infectious diseases register.

The number of hospitalised cases (n = 129) is presented in bars with Y-axis on the left and the number of positive adenovirus tests reported to NIDR (n = 888) is presented with grey shaded area with Y-axis on the right. The total number of adenovirus tests performed was not available.

Civilian contacts include close contacts of conscripts (n = 19), cases with possible epidemiological link to conscripts (n = 9) and healthcare-associated infections (n = 4).

In February and early March, all cases were linked to a single garrison. Later, the epidemic spread to six other garrisons mainly in southern Finland, where also the first affected garrison is located. In all, conscripts in basic military training in Finland are housed in 18 garrisons.

Median age of the hospitalised cases was 20 years (interquartile range (IQR): 19–33; range: 0–90), 81.4% were male (105/129) and 18.6% were female (24/129). The majority of those with clinical data available were previously healthy (66/98; 67.3%). Only 12.2% (12/98) had complex underlying medical conditions, and 5/98 (5.1%) were immunocompromised. Civilians were older and more frequently had underlying medical conditions compared to the conscripts (Table 1).

In all cases, adenovirus was detected in respiratory samples, and in most cases, samples were tested using syndromic PCR panels. Adenovirus was the sole pathogen detected in 84 of 120 (70.0%) of the cases with data available. The most common pathogens co-detected with adenovirus were rhinovirus/enterovirus (9/120; 7.5%) followed by seasonal coronavirus (7/120; 5.8%), parainfluenza virus 3 (7/120; 5.8%) and *Mycoplasma pneumoniae* (7/120; 5.8%) (Table 1).

Cases typically presented with fever and respiratory symptoms. The clinical presentation of more severe cases was complicated by respiratory failure, encephalitis and hepatitis. Of the 129 hospitalised cases, 99 (76.7%) were admitted to a normal ward and 30 (23.3%) to the ICU (Table 1 and 2). Ten (7.8%) cases required ECMO. Median length of hospital stay was 4 days (IQR: 3–6) in all cases and 12 days (IQR: 8–25) for cases admitted to the ICU (data were available for 84/129 cases). Six (4.7%) civilian cases died, five of whom had contact with conscripts (Table 1). There were no deaths in conscripts. Of the deceased cases, four of five were immunocompromised and/or had a complex underlying medical condition. 

**Table 2:** Characteristics of cases hospitalised with adenovirus infection stratified by outcome severity, Finland, 1 February–30 June 2024 (n = 129)

Characteristics	Normal ward		ICU^a^		ECMO^a^		Deceased^a^		Total		p^b^
	n = 99		n = 30		n = 10		n = 6		n = 129		
	n	%	n	%	n	%	n	%	n	%	
Age in years, median (IQR)	20 (19–21)		24 (19–51)		42 (21–52)		53 (47–62)		20 (19–33)		0.0296
Sex											
Male	81	81.8	24	80.0	7	70.0	4	66.7	105	81.4	0.79
Female	18	18.2	6	20.0	3	30.0	2	33.3	24	18.6	
**Underlying medical conditions **											
No underlying medical condition	54	69.2	12	57.1	3	60.0	1	20.0	66	66.7	0.07^c^
Non-severe chronic medical condition	12	15.4	3	14.3	0	0	0	0	15	15.1	
Complex or ≥ 2 chronic medical conditions	9	11.5	4	19.0	0	0	1	20.0	13	13.1	
Immunocompromised	3	3.8	2	9.5	2	40.0	3	60.0	5	5.1	
Data not available	21		9		5		1		30		
**Co-detection of other viruses or bacteria **											
No other pathogens	63	67.0	21	80.8	3	50.0	2	40.0	84	70.0	0.23^d^
One additional pathogen	26	27.7	4	15.4	2	33.3	1	20.0	30	25.0	
Multiple additional pathogens	5	5.3	1	3.8	1	16.7	1	20.0	6	5.0	
*Bordetella pertussis*	2	2.1	1	3.8	1	16.7	0	0	3	2.5	
*Mycoplasma pneumoniae*	5	5.3	2	7.7	2	33.3	1	20.0	7	5.8	
*Streptococcus pyogenes*	1	1.1	0	0	0	0	0	0	1	0.8	
Influenza A	0	0	0	0	0	0	0	0	0	0	
Influenza B	0	0	1	3.8	0	0	0	0	1	0.8	
Metapneumovirus	3	3.2	0	0	0	0	0	0	3	2.5	
Parainfluenza 3 virus	7	7.4	0	0	0	0	0	0	7	5.8	
Parainfluenza virus (type unknown)	2	2.1	0	0	0	0	0	0	2	1.6	
Rhino/enterovirus	5	5.3	1	3.8	1	16.7	1	20.0	9	7.5	
RSV	1	1.1	0	0	0	0	0	0	1	0.8	
SARS-CoV-2	2	2.1	0	0	0	0	0	0	2	1.6	
Seasonal coronavirus	6	6.4	1	3.8	0	0	1	20.0	7	5.8	
Data not available	5		4		4		1		9		
**Adenovirus type **											
Type 7	70	87.5	27	96.4	10	100	5	100	97	89.8	0.29^f^
Type 14	0	0	1	3.6	0	0	0	0	1	0.9	
Negative^e^	10	12.5	0	0	0	0	0	0	10	9.3	
Data not available	19		2		0		1		21		

ECMO: extracorporeal membrane oxygenation; IQR: interquartile range; ICU: intensive care unit; RSV: respiratory syncytial virus; SARS-CoV-2: severe acute respiratory syndrome coronavirus 2.

^a^ All ECMO-treated and deceased cases were admitted to the ICU and are also included in the ICU strata.

^b^ Statistical comparisons were performed between cases admitted to normal ward vs ICU. Mann–Whitney test was used to compare continuous variables and Chi-square or Fisher’s exact test to compare proportions.

^c^ Comparison between no underlying medical condition vs any underlying medical condition or immunosuppression.

^d^ Comparison between no other pathogens vs one or more additional pathogens.

^e^ PCR typing for adenovirus types 3, 4, 7, 11, 14, 16 and 21 was negative.

^f^ Comparison between adenovirus type 7 vs type 14 or adenovirus typing negative.

In response to the outbreak, infection control measures at garrisons were intensified, including enhanced hand hygiene, enhanced cleaning of toilets, surfaces, door handles and other touchpoints, the use of face masks and cohorting of symptomatic conscripts. Measures were enhanced gradually, and all were implemented by 30 April.

## Molecular characterisation of adenovirus

Of the 129 hospitalised cases, samples were available for typing from 108 cases (83.7%). Samples were typed by PCR for adenovirus types 3, 4, 7, 11, 14, 16 and 21 [2,3]. Of the typed samples, 97 of 108 (89.8%) were type 7, one of 108 (0.9%) was type 14 and 10 of 108 (9.3%) were negative for these types. In conscripts and their contacts, the proportion of adenovirus type 7 was above 95%, whereas in cases with no contact with conscripts, only nine of 17 (52.8%) samples were type 7.

**Figure 2:**
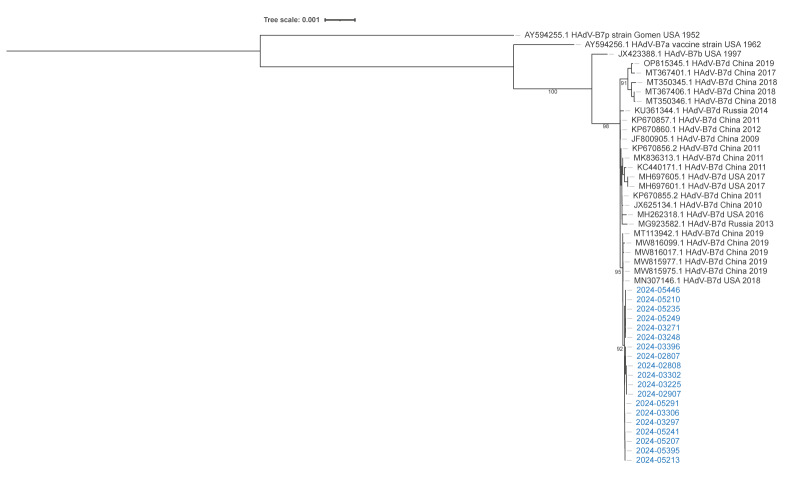
Phylogenetic analysis of historical adenovirus sequences (n = 27) with adenovirus sequences from the current outbreak (n = 19), Finland, 1 February–30 June 2024

Whole genome sequencing of 24 adenovirus samples confirmed the virus type to be 7d. Close clustering of the outbreak viruses was observed in a phylogenetic tree displaying Finnish and global subtype 7d adenovirus sequences (Figure 2). The hexon genes were identical and the whole genomes differed by 0–3 nucleotides in the non-coding and inverted terminal repeat regions between the Finnish sequences. The distance between Finnish and global reference sequences was 2 or more nucleotides. Two synonymous mutations (10 049A and 27 797G) were unique to the Finnish sequences. Overall, the genomes appear highly conserved based on the high sequence identities and modest divergence from the closest sequences from 2009 onwards from China, Russia and the United States (US) (Figure 2).

HAdV: human adenovirus.

Whole genome sequencing was performed using the Illumina Respiratory Virus Enrichment kit (Illumina). The reference sequences (n = 27) were chosen from BLAST results and sequences from previously published studies, including the HAdV vaccine strain (AY594256.1) and an early isolate as outgroup (AY594255.1). High quality sequences from the current outbreak (n = 19) and relevant reference sequences were aligned with MAFFT (v. 7.535) and the maximum likelihood tree was generated with IQ-TREE web server (http://iqtree.cibiv.univie.ac.at). All the sequences presented in the tree are publicly available in GenBank. The tree was visualised with iTOL (https://itol.embl.de).

## Register-based case detection from 1 August–30 November 2024

The active data collection was discontinued in July as the outbreak began to subside. Since August 2024, monitoring of adenovirus infection has been based on national registry data. The number of cases hospitalised with a laboratory-confirmed adenovirus infection or an adenovirus-specific ICD-10 code began to rise at the end of August, peaked in September–October and then declined again.

From 1 August to 30 November 2024, a total of 274 cases were hospitalised, of which 43 (15.7%) were admitted to the ICU. Of the 274 cases, 65 (23.7%) were conscripts. The typing result was available for 157 cases: 96 (61.1%) were type 7 and 57 (20.8%) type 4. The proportion of other adenovirus types and samples without typing data increased towards November. Among ICU cases, 24/27 (88.9%) were adenovirus type 7. Because of the data collection method and registration delays, we do not have information on civilians’ contacts to conscripts and information on the number of deaths.

## Discussion

Adenoviruses circulate in the community every year. Adenoviruses that infect humans are classified into seven species (mastadenovirus A to G) that comprise over 110 recognised genotypes [4,5]. Adenovirus outbreaks are known to occur in closed settings including healthcare units, daycare centres, long-term care facilities and military training centres. Adenovirus types 4, 7 and 14 are the types most often responsible for outbreaks in garrisons or other settings with close contacts [6-11]. 

Adenovirus type 7, especially subtype 7d strains, have been associated with severe clinical presentation [9-13]. In an outbreak in Oregon, US in 2013–14, type 7d was the dominant strain and ICU admission rates in hospitalised patients reached 46% [14]. It is likely that the severe outbreak reported here is largely explained by the severe disease associated with adenovirus type 7d. 

In a surveillance study covering several Finnish garrisons in 2008–12, adenoviruses were detected every winter season [15]. The majority of positive samples were type 4 (487/837; 58.2%) followed by type 3 (181/837; 21.6%), while type 7 strains were not detected at all. At the HUS Diagnostic Center between 2002 and 2018, a total of 619 adenovirus-positive samples were typed, and only 18 (2.9%) were type 7 (unpublished data). Re-emergence of adenovirus type 7d was reported in Asia in 2009 after a hiatus of 21 years [16]. In the US, type 7d strains have been detected since 2013 [13], but there are no previous reports of outbreaks caused by this subtype in Europe. Therefore, another potential factor contributing to the unusually severe outbreak is that type 7 strains likely have not circulated in Finland over a long period of time and the pre-existing population-level immunity may have been low.

In Finland, every male citizen aged 18–60 is liable for military service. Women can apply on a voluntary basis. Military training is done between ages 18–30 years, lasts 165–347 days, and is provided annually for ca 21,000 conscripts. Every year, a new group of conscripts start their service in January or July. During service, conscripts live in close quarters, typically nine conscripts sharing a room, but are regularly on home-leave and therefore an integral part of society. 

In the US, an oral live unattenuated adenovirus vaccine against types 4 and 7 is available and FDA approved for military use among those 17 to 50 years of age. The current vaccine has been used to vaccinate US military recruits and personnel since 2011 [8,17-19]. The vaccine is not used in civilians, and it is not available outside the US.

In the spring outbreak in Finland, only limited spread and sporadic cases were detected in the general population. This is in line with previous observations [7] and it seems likely that the adenovirus type 7, along with other strains capable of causing outbreaks in settings with close contacts, are usually associated with only limited spread in the community. In contrast, in the autumn epidemic, the general population was more affected, possibly because of an increase in other circulating adenovirus types.

The limitations of this report include the lack of data on the total number of adenovirus- detecting tests performed, which prevents us from estimating the test positive percentage at the national level. Even if we collected information on civilian cases contacts with conscripts and garrisons, we did not specifically collect data on contacts between conscript cases and civilian cases. As the active case detection was discontinued in July, we were unable to combine the data from the cases detected before July and those identified from registries since August 2024.

## Conclusion

The current outbreak highlights the risk adenovirus poses to individuals living in closed settings, immunocompromised individuals and those with underlying illnesses as well as the risk for healthcare associated infections. Factors that may have contributed to the severity of the current outbreak include close contacts between military conscripts, potential lack of immunological memory and the capability of adenovirus type 7d to cause severe disease. Increased surveillance efforts, better understanding of the circulating adenovirus strains, and early response will be key elements in controlling future outbreaks. Also, the possibility of licensing the live adenovirus vaccine in the European Union should be explored.
